# Minilifting: Short-Scar Rhytidectomy with Thread Lifting

**DOI:** 10.1055/s-0044-1788907

**Published:** 2024-09-27

**Authors:** Kyu Hwa Jung, Won Lee

**Affiliations:** 1 Liting Plastic Surgery Clinic, Seoul, South Korea; 2 Yonsei E1 Plastic Surgery Clinic, Anyang, South Korea

**Keywords:** minilifting, thread lifting, PDO lifting

## Abstract

Facelifting techniques have been developed over time to mask the aging process. However, conventional facelifts cause scarring. Because of patient demands, various noninvasive lifting techniques have been introduced, including absorbable thread lifting. Minilifting is known for its short-scar excision and is used to improve skin laxity and lifting using absorbable threads but the definition and operation techniques are not certain. In this article, we described the definition, development, and operative techniques used in minilifts. Minilifting procedures represent an added option for patients with minimal scarring and adequate lifting effects.

## Introduction

The demand for maintaining a youthful appearance has been a reality for a long time, and the demand for cosmeceutical products is increasing. 1 Centuries ago, Cleopatra was said to bathe in sour milk in order to give her skin a youthful appearance. 2 However, with the development of science, more evidence-based antiaging methods have been introduced. 3 Facial antiaging surgical procedures have also been developed, starting with subcutaneous facelift, which is the excision of loosened skin at the subcutaneous level. 4 5 Facial anatomical knowledge has also developed, allowing for a better understanding of the superficial musculoaponeurotic system (SMAS). 6 7 8 9 There have also been multiple studies on the facial retaining ligaments, 10 11 12 and they showed that a greater lifting effect can be seen when these retaining ligaments are released. 13 More invasive techniques have been suggested to achieve greater lifting effects. 12 13 However, when surgical techniques are more invasive, a greater fear can be observed in patients. 14 Therefore, less invasive methods have been developed. Multiple investigations of short-scar rhytidectomy have been performed to reduce scars and complication risks. 15 16 17 18 19 20 21 However, from the patient's perspective, these techniques are still invasive facelift procedures. 22

Noninvasive thread lifting was introduced in the early 1990s. 23 Subsequently, multiple threading methods were developed, and various techniques were introduced. 24 25 26 Noninvasive thread-lifting techniques have greatly increased their presence in the aesthetic market. 27 However, the effectiveness of thread lifting is questionable, suggesting that the lifting effect could disappear after a short time owing to thread migration in the temporal area. 28 Therefore, another solution that combines minimal invasiveness and maximal lifting effects should be developed. Accordingly, minilifting has been developed to reduce invasiveness and improve patient satisfaction. 29 30

## Definition

“Minilifting” has been described as a procedure involving relatively small incisions that excise skin to remove wrinkles in specific areas. 31 However, there was a lack of understanding regarding the SMAS; therefore, only skin excision was performed initially. 31 After further research, facelifts involving the SMAS resulted in improved facial contouring with skin tightening through skin excision. 32 33 Minimally invasive facelifts with small incisions and facial contouring have also been introduced. 15 16 17 However, minimal invasive facelift, short-scar rhytidectomy, and “minilifting” have been introduced as similar techniques that share preauricular incisions. 34 Thus, the “minilifting” technique can be defined as the excision of skin for skin tightening within or at the hairline and thread lifting for facial contouring by superficial fat repositioning.

## Development

In the early 1900s, Passot introduced the method of excising the remnant skin to improve wrinkles. However, these procedures are limited to skin excision and scar problems due to excessive skin tension. 31 However, minilifts have been in the spotlight for a long time due to the resulting midface rejuvenation. Tessier introduced a midface lift using the subperiosteal approach to overcome the limitations of the conventional midface effect. 35 Since then, various subperiosteal approaches to perform deep layer suspension have been introduced. 36 37 38 39 In addition, a subperiosteal approach has been developed for a more invisible hairline incision, 40 and even incision scars are made smaller using an endoscope. 41 The subperiosteal approach, which is the deepest layer suspension technique, can have a longer lifting effect but it has limitations in improving the nasolabial fold. 39 It has been revealed that cutaneous lifts with SMAS management have a more dramatic facelift effect. 42

Thus, the concept has changed from the subperiosteal elevation of the malar fat pad to its direct suspension on a limited movable area. 43 Su introduced the “closed suspension mini-cheek lift,” which consists of the fixation of the malar fat pad to the deep temporal fascia (DTF) by a percutaneous approach. 44 Sasaki and Cohen introduced a midface lift using a cable suture technique with Gore-Tex (polytetrafluoroethylene) and Vicryl (polyglactin). 45 As this suture technique has proven effective and long-lasting, multiple percutaneous midface lifting techniques have since been introduced. 46 47 Midface rejuvenation by nonabsorbable sutures was introduced but has limitations in the use of the ancillary procedure of conventional facelifts. 48 This has also been suggested for problems with nonabsorbable sutures. 49

Since then, absorbable threads have been used. “Lunchtime lifting,” marked by the use of polydioxanone (PDO)-barbed thread, was introduced; since then, PDO thread-lifting has been a trend in the antiaging market because of its lower risk of complications. 50 51 However, the effectiveness of nonabsorbable thread lifting is also questionable compared to the patient's expectations. 52 Consequently, minilifting using a PDO-barbed thread has been introduced. 53

## The Concept

During the aging process, soft tissue descends owing to the loss of tissue-supporting structure volume and sagging due to gravity. 54 Minilifting is a tissue repositioning procedure that uses the percutaneous approach of a biomaterial thread. 55 Excessively sagged skin can be improved either by fat tissue repositioning, which is firmly attached to the skin, or by expansion effect by volume restoration. 47 56 Long-term effectiveness can be secured by fixing the thread to a firm tissue. 47 57 The minilifting concept involves facilitating sufficient mobility of the skin by dissection; thus, the target tissue can be easily repositioned using a thread. Undermining soft tissues can eliminate skin pleating and puckering, which can only be achieved by thread lifting. To solve the problem of skin pleating and puckering, the amount of repositioned tissue should be reduced or skin excision should be performed. 57 58 In conclusion, minilifting requires a short recovery time due to minimal dissection but can redistribute skin tension so that it has a greater lifting effect than traditional thread lifting.

## Indications

The selection of a good minilifting candidate is one of the most important factors for patient satisfaction. 57 Sasaki and Cohen described that an ideal candidate is a 35- to 45-year-old patient with early midface laxity primarily involving ptosis of the malar fat pad. 45 Pontius and Williams suggested the 40- to 50-year range but considered a concomitant browlift procedure. 58 Quatela and Antunes reported that minilifts are not suitable for patients with severe facial volume loss. 57 According to Laferriere and Castellano, the procedure can be helpful in older patients with significant midface ptosis when performed as an open procedure, allowing for a more conservative lower facelift. 47 In conclusion, the procedure is suitable for patients with mild-to-moderate midface ptosis, a mildly descending lower face, and no severe skin laxity or volume loss. This procedure can be applied to male patients or patients undergoing a secondary facelift. 46 Midface rejuvenation and improved lower face techniques have been introduced; thus, additional indications can be applied. 59

## Operative Design

### Incision

**Fig. 1:**
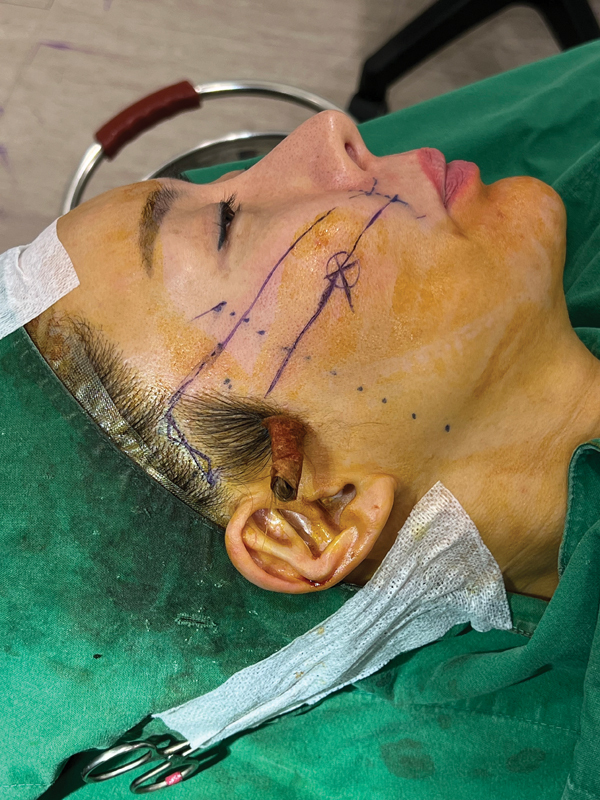
Minilifting posterior hairline incision. A zigzag incision of 2 to 3 cm inside the hairline is visible. The upper line is a virtual line between the point of the nasolabial fold and alar base to 1 cm lateral to the lateral orbital rim. The lower line is a virtual line from 1 cm lower to the nasolabial fold and alar base to the zygomatic ligament area.

A surgical incision line is usually made with a length of 3 to 4 cm at 1 to 2 cm posteriorly from the hairline, which corresponds to the superior area of the temporalis muscle and the area between the inferior and superior temporal lines. 45 46 47 50 57 58 A prehairline incision and a posterior hairline incision can be made concomitantly. 60 The authors prefer to perform a posterior hairline incision because patients who decide to undergo minilifting usually prefer not to have a visible scar and have a short recovery time ( Fig. 1 ).

### Insertion Point

**Fig. 2:**
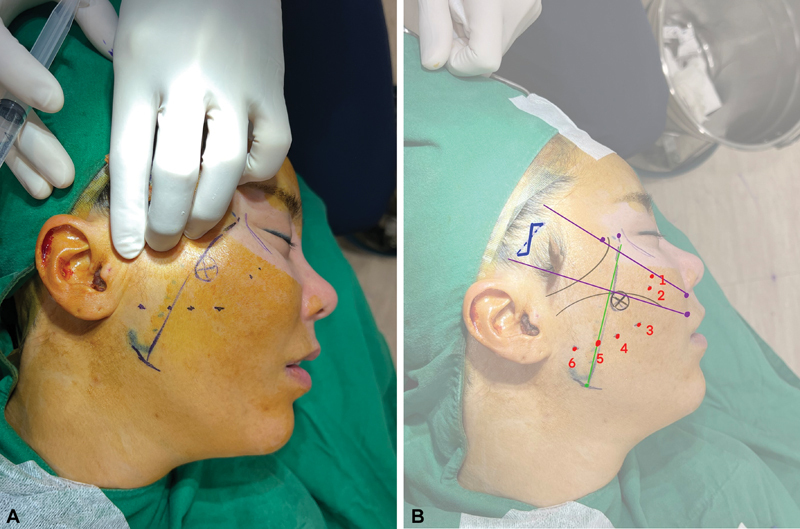
Schematic diagram of insertion points. ( **A** ) Pulling the skin and designing four points located from the otobasion inferius to the subnasale. ( **B** ) Four points were made (3–6). Points 3 and 4 are for jowl fat compartment lifting and points 5 and 6 are for lateral cheek fat compartment lifting. Points 1 and 2 are for the most projecting area after pinching the arcus marginalis and zygomatic ligament area.

Sasaki and Cohen used a nasolabial crease insertion point at the level of the alar base, and a second insertion point was made 1 cm lower, lateral to the first insertion point, and made a 1.5-cm incision 1.0 cm behind the temporal hairline 45 ; Laferriere and Castellano described the same insertion point. 47 The malar fat pad anchoring point can be made more lateral than that in previous studies. 59 Another study described the target malar fat pad anchoring point as 2 cm lower than the lateral canthus. 61 This area is located between the arcus marginalis and the zygomatic ligament. Using these two points, an insertion point can be created at the projected malar fat pad after pinching. 51 Another suggestion for insertion points at the alar base and otobasion level is concomitant lower face lifting. 53 The authors usually perform pinching at the arcus marginalis and zygomatic ligament. The two most projected points are marked in Fig. 2 . Additionally, four points located at the virtual line of the alar base are used to avoid obstruction after pulling the upper skin with the left hand ( Fig. 2 ).

### Anesthesia

Usually, minilifting uses local anesthetic infiltration with or without sedation using diazepam (10 mg or 0.5 mg/kg) or intravenous sedation but general anesthesia can also be performed. 48 51 59 Local anesthesia can be achieved with 1% xylocaine with 1:400,000 epinephrine and a regional nerve block with 0.5% Marcaine (bupivacaine HCl; 1:200,000 epinephrine) at the zygomaticofacial, zygomaticotemporal, and infraorbital nerves. 45 Other articles have described the use of 10 to 20 mL of 2% lidocaine with 1:100,000 epinephrine. 61 A tumescent solution (0.3% lidocaine, 1:650,000 epinephrine, 2 mEq of sodium carbonate) can be used; however, when a PDO thread is used for lifting, the use of a tumescent solution is not recommended because of degradation. 50 The authors prefer intravenous sedation by propofol and local anesthesia using 2% lidocaine because a tumescent solution can degrade the PDO thread sooner than expected.

### Procedure

Most procedures involve surgical dissection following a temporal incision. Dissection can be performed between the superficial temporal fascia (STF) and DTF. 45 46 47 58 Dissection can be made through the subcutaneous layer, which is superficial to the STF. 61 Tarallo et al performed dissection at the subcutaneous layer for skin flap elevation, followed by additional dissection at the sub-SMAS layer. 60 After dissection, most techniques involve fixation at the DTF and use either absorbable or nonabsorbable barbed sutures. 50 53

**Fig. 3:**
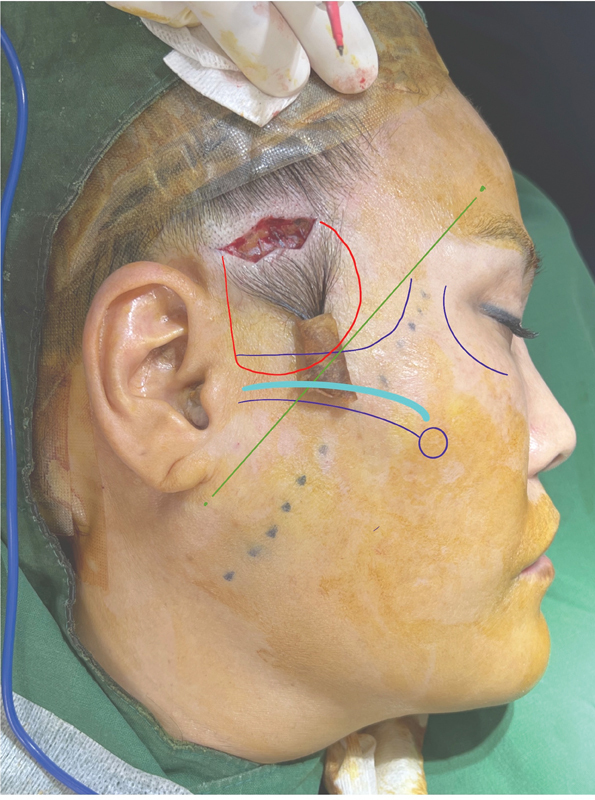
Incision and subcutaneous dissection. Dissection border (red line), Pitanguy's line (green line), and lateral zygomaticocutaneous ligament (sky blue line).

Our technique involves a zigzag incision on the hairline. Subcutaneous dissection was then performed until Pitanguy's line was reached medially and the zygomatic arch upper border was reached caudally, using Metzenbaum scissors or finger dissection to perform a partial release of the zygomaticocutaneous ligament ( Fig. 3 ).

**Fig. 4:**
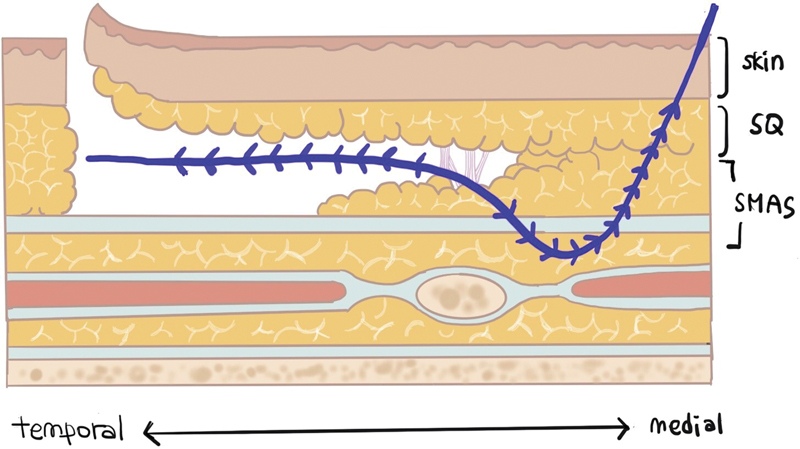
Thread insertion layer. From the insertion point, threads should be inserted deeply to hold the SMAS layer and should exit at the incision space. SMAS, superficial musculoaponeurotic system; SQ, subcutaneous layer.

Six insertion points were made using an 18G needle ( Fig. 2 ). Cannula-bidirectional-cogged 19G 150-mm PDO threads, USP 0 (N cog; N Finders Co., Seoul, South Korea), were used for the reverse technique. Threads were inserted deeply to hold the SMAS layer and exit the incision site ( Fig. 4 ).

**Fig. 5:**
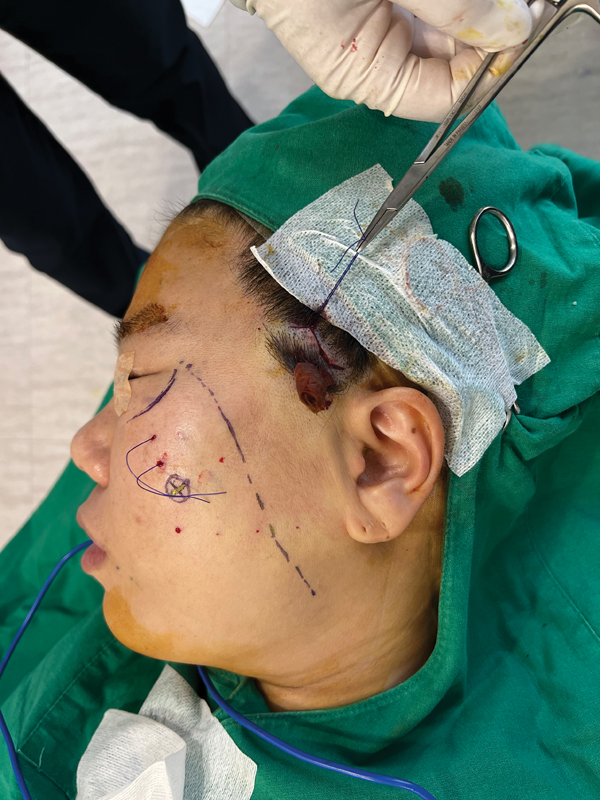
Thread insertion and loop formation with two threads. Fixation at the deep temporal fascia is performed, and the lifting effect in the surface anatomy can be seen.

Two adjacent threads are held together at the incision site to form a loop. The thread inserted at point 1 should be held at point 2 (3 at 4 and 5 at 6), and the two threads should be tied. The repositioned tissue should be checked to ensure excessive tension is not induced by pulling the threads ( Fig. 5 ). Fixation was performed at the DTF using a long needle 2–0 PDO. Subcutaneous sutures were made using PDO 4–0, and skin sutures were made using nylon 5–0.

## Longevity

**Fig. 6:**
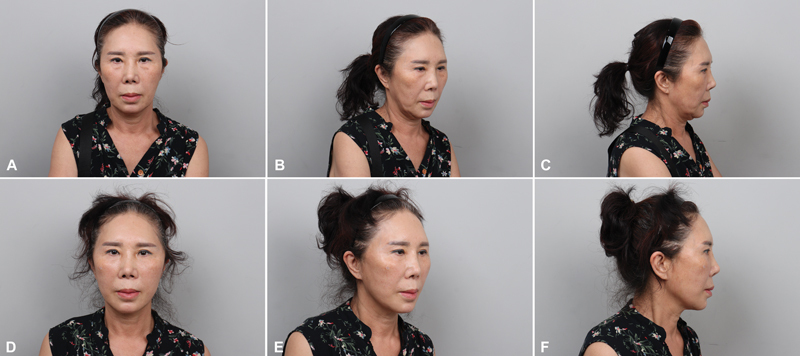
A 66-year-old woman before and after minilifting. ( **A** ) Preoperative frontal view. ( **B** ) Preoperative three-quarter view. ( **C** ) Preoperative lateral view. ( **D** ) Postoperative 1-month frontal view. ( **E** ) Postoperative 1-month three-quarter view. ( **F** ) Postoperative 1-month lateral view.

Hamra reported that even in patients with a deep plane facelift, the midface repositioning effect was prolonged for 1 to 2 years. 62 Sasaki and Cohen reported that patient satisfaction was up to 85% 2 years after minilifting. 45 Keller et al reported that the effect was prolonged for 1 year. 46 Laferriere and Castellano reported that the lifting was maintained for 1 to 2 years in a 28-month follow-up study, 47 where Gualdi et al found that the lifting effect was prolonged for a year using nonabsorbable sutures. 50 According to the authors' experience, the longevity of minilifts is 1 year ( Fig. 6 ). However, the tensile strength of the PDO diminishes by up to 60% at 3 months after insertion; thus, the lifting effect, as seen immediately after the operation, is not prolonged for a year. However, when minilifting is performed two to three times, the prevention of tissue sagging seems to last longer according to our experience. It seems that not only mechanical effects can be performed by PDO thread but also chemical effects such as collagenesis might be performed. 53

## Complications

**Fig. 7:**
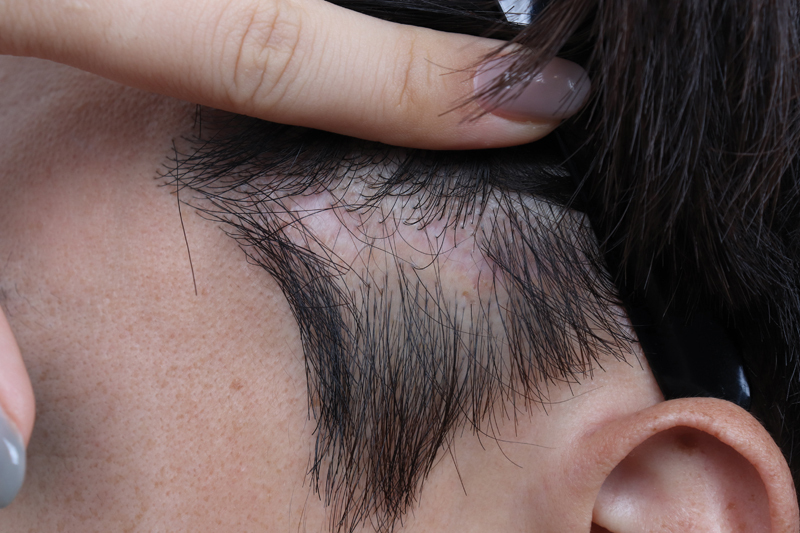
A 42-year-old man 1 month after minilifting. Scar revision coverage with a cephalic flap was performed.

Minor lifting complications include asymmetry, skin dimples, motor weakness, and infection. 43 Asymmetry and skin dimples usually resolve naturally within 3 months. Surgical revision may be performed to correct the asymmetry but it usually resolves without surgery. 44 The authors have also experienced complications such as temporal pain and neuropraxia, which resolved spontaneously. Alopecia may also develop but can resolve with or without surgical correction ( Fig. 7 ). Surgical correction can be a direct excision of alopecia.

## Conclusion

Conventional facelift techniques have been in use for decades. However, patient demand for noninvasive techniques and more effective procedures is increasing. Minilifting can be an alternative for patients who expect minimal scarring and a significant lifting effect.
